# Specific detection of soluble EphA2 fragments in blood as a new biomarker for pancreatic cancer

**DOI:** 10.1038/cddis.2017.545

**Published:** 2017-10-26

**Authors:** Naohiko Koshikawa, Tomoko Minegishi, Hirofumi Kiyokawa, Motoharu Seiki

**Affiliations:** 1 Division of Cancer Cell Research, Kanagawa Cancer Center Research Institute, Yokohama, Japan; 2 Division of Cancer Cell Research, Institute of Medical Science, University of Tokyo, Tokyo, Japan; 3 Division of Gastroenterology and Hepatology, St. Marianna University School of Medicine, Kawasaki, Japan; 4 Faculty of Medicine, Institute of Medical, Pharmaceutical, and Health Sciences, Kanazawa University, Kanazawa, Japan

## Abstract

Because membrane type 1-matrix metalloproteinase 1 (MT1-MMP) and erythropoietin-producing hepatocellular receptor 2 (EphA2) expression are upregulated by the Ras/mitogen-activated protein kinase pathway, they are frequently coexpressed in malignant tumors. MT1-MMP cleaves the N-terminal ligand-binding domain of EphA2 and inactivates its ligand-dependent tumor-suppressing activity. Therefore, specific detection of the cleaved N-terminal EphA2 fragment in blood might be an effective biomarker to diagnose malignant tumors. To evaluate this possibility, we developed three monoclonal antibodies against the soluble EphA2 fragment. One of them recognized this fragment specifically, with negligible cross-reactivity to the intact form. We used the cleaved form-specific antibody to develop a quantitative enzyme-linked immunosorbent assay and confirmed the linear reactivity to the recombinant fragment. We applied this assay on commercially available serum specimens obtained from patients with several types of cancer including gastric, pancreatic, esophageal, gastroesophageal, and head-and-neck cancers, and healthy donors. Soluble EphA2 fragment levels in cancer-patient sera were higher than those in healthy donors (*n*=50). In particular, levels of eight out of nine (89%) pancreatic cancer patients and ten out of seventeen (59%) gastric cancer patients significantly exceeded cutoff values obtained from the healthy donors, whereas those of esophageal and head-and-neck cancer-patient sera were low. The preliminary receiver operating characteristic curve analysis for pancreatic cancer demonstrated that the sensitivity and specificity were 89.0% and 90.0%, respectively, whereas those of the conventional digestive tumor marker CA19-9 were 88.9% and 72.0%, respectively. These results indicated that specific detection of soluble EphA2 fragment levels in serum could be potentially useful as a biomarker to diagnose pancreatic cancer.

Erythropoietin-producing hepatocellular receptor 2 (EphA2), a member of the mammalian Eph receptor kinase family, is expressed in epithelial cells and acts to suppress unscheduled growth signals for stabilization of the epithelial cell phenotype. In addition, EphA2 is expressed in vascular endothelial cells and has a role in tumor vessel formation through the stimulation of cell migration.^1, 2^ Furthermore, it is also overexpressed frequently in different types of cancers such as breast, liver, pancreatic, prostate, esophageal, glioma, melanoma, and ovarian adenocarcinoma.^3^ Because overexpression of EphA2 is associated with disease progression and metastasis of cancer cells, and it is thought to be a possible target for cancer therapy,^3, 4^ small-molecule inhibitors, siRNAs, and neutralizing antibodies against EphA2 are currently under evaluation while leaving the possibility of EphA2 as a tumor suppressor.

Recently, it became clear that EphA2 regulates ErbB receptor-mediated signals differently depending on availability of its ligands, ephrins. In the presence of ephrins, EphA2 suppresses ErbB receptor signals, whereas ligand-independent EphA2 signals cooperate with ErbB receptor-mediated signals to promote tumor growth and invasion.^5^ Therefore, the availability of ligands appears to determine which function of EphA2 predominates during tumor progression.

EphA2 ligands (for example, ephrin-A1) are expressed abundantly in different tumor types such as breast carcinoma and melanoma, and normal tissues.^6^ Soluble ephrin-A1 is released by cleavage of matrix metalloproteinases (MMPs) and is detected in the sera of patients with hepatocellular carcinoma.^7, 8^ Therefore, critical questions are how and when ligand-independent EphA2 signaling is activated in tumors.

We have demonstrated recently a possible mechanism involving proteolytic processing to convert EphA2 into a ligand-insensitive form.^9, 10, 11^ Membrane type 1-MMP (MT1-MMP) is a membrane MMP that is frequently overexpressed in malignant tumors. MT1-MMP regulates tumor progression via processing of bioactive proteins and extracellular matrices on the cell periphery, as well as through activation of hypoxia-inducible transcription factors in a cytoplasmic tail-mediated manner.^12, 13, 14^ The expression of both EphA2 and MT1-MMP is upregulated by the Ras/mitogen-activated protein kinase (MAPK) pathway and is frequently observed on cancer cell membranes.^15, 16^ EphA2 is an MT1-MMP substrate. Its cleavage by MT1-MMP eliminates the ligand-binding domain and converts it into a ligand-unavailable form by inactivating its tumor-suppressor activity.^9, 10^

Immunohistochemical analysis of several cancer tissues revealed that EphA2 coexpressed with MT1-MMP in several carcinoma tissues mostly lacked the N-terminal ligand-binding domain, and activated ligand-independent EphA2 signaling (phosphorylation of Ser^897^).^5, 9^ Forced expression of an uncleavable EphA2 mutant in carcinoma cells altered cell morphology to epithelial-like forms, and suppressed tumor growth and lung metastasis in mice. Therefore, processing of EphA2 by MT1-MMP in cancer cells represents an important cancer progression-related event. If the cleaved N-terminal domain of EphA2 is released from cells and circulates stably in patient blood, it could be a useful biomarker for early detection in cancer patients using a conventional blood test. During preparation of this manuscript, another group reported that serum EphA2 could be a potential biomarker to diagnose pancreatic cancer, although that report made no mention of the form of EphA2.^17^

In the current study, we developed monoclonal antibodies (mAbs) against the soluble EphA2 fragment released from cancer cells by MT1-MMP cleavage. Several clones were obtained, with one showing reactivity to the soluble EphA2 fragment and negligible cross-reactivity to the intact form. We used this fragment-specific antibody to develop a sandwich enzyme-linked immunosorbent assay (ELISA) and applied it to measure levels of the soluble EphA2 fragment in sera from cancer patients.

## Results

### Purification of recombinant antigens

**Figure 1:**
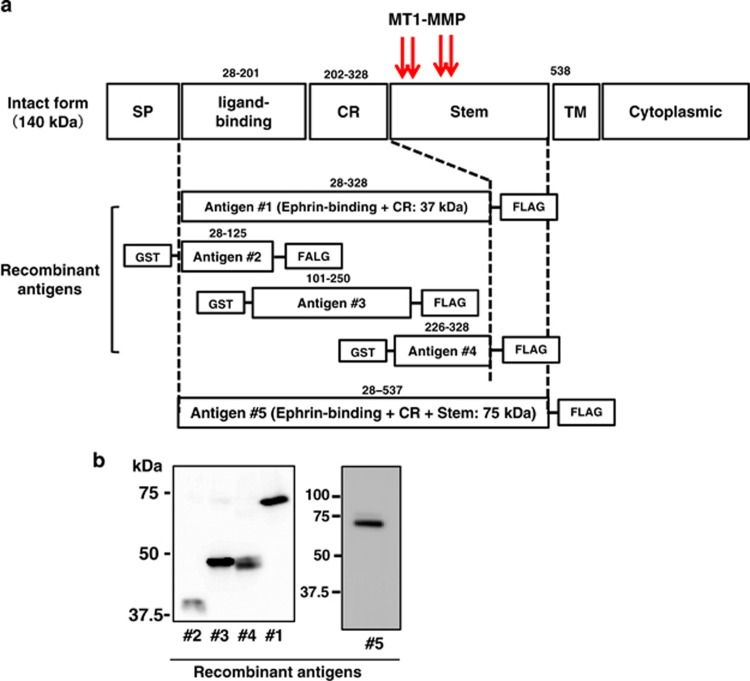
Preparation of recombinant EphA2 and its fragments. (**a**) Intact EphA2, signal peptide (SP), ligand-binding, cysteine-rich (CR), stem, transmembrane (TM), and cytoplasmic domains are denoted. Red arrows indicate the cleavage sites by MT1-MMP. Recombinant antigens #1 and 5 were expressed with the C-terminal FLAG-tag in FreeStyle 293-F or A431 cells as secreted proteins in conditioned medium. Antigens #2, 3, and 4 were produced in *Escherichia coli*. (**b**) All antigens were analyzed by western blot using an anti-FLAG mAb under reducing conditions after purification on an anti-FLAG mAb-conjugated agarose column. GST: glutathione S-transferase

The extracellular portion of EphA2 following the signal peptide (1–27 amino acids (aa)) is composed of the ligand-binding domain (28–201 aa), the cysteine-rich domain (202–328 aa), and the stem region (329–537 aa) from the N terminus (Figure 1a). We and others mapped the cleavage sites of EphA2 by MT1-MMP in the stem region as indicated by arrows in Figure 1a.^9, 18^ For immunization to raise mAbs, we prepared recombinant antigens spanning 28–328 aa (antigen #1, 75 kDa) and 28–537 aa (antigen #5, 75 kDa) by expressing, respectively, the 1–328 and 1–537 regions in FreeStyle 293-F (Thermo Fisher Scientific, Waltham, MA, USA) and A431 cells as FLAG-tagged forms at the C terminus (Figure 1a). The antigens were purified and confirmed by western blotting using an anti-FLAG mAb. We prepared three additional FLAG-tagged antigens (#2, 3, and 4; 40, 50, and 50 kDa, respectively) by expressing them in *Escherichia coli* as fusion proteins with an N-terminal glutathione S-transferase tag. Each antigen was purified and detected as before (Figure 1b).

### Reactivity of mAbs against the cleaved form of the EphA2 fragment

**Figure 2:**
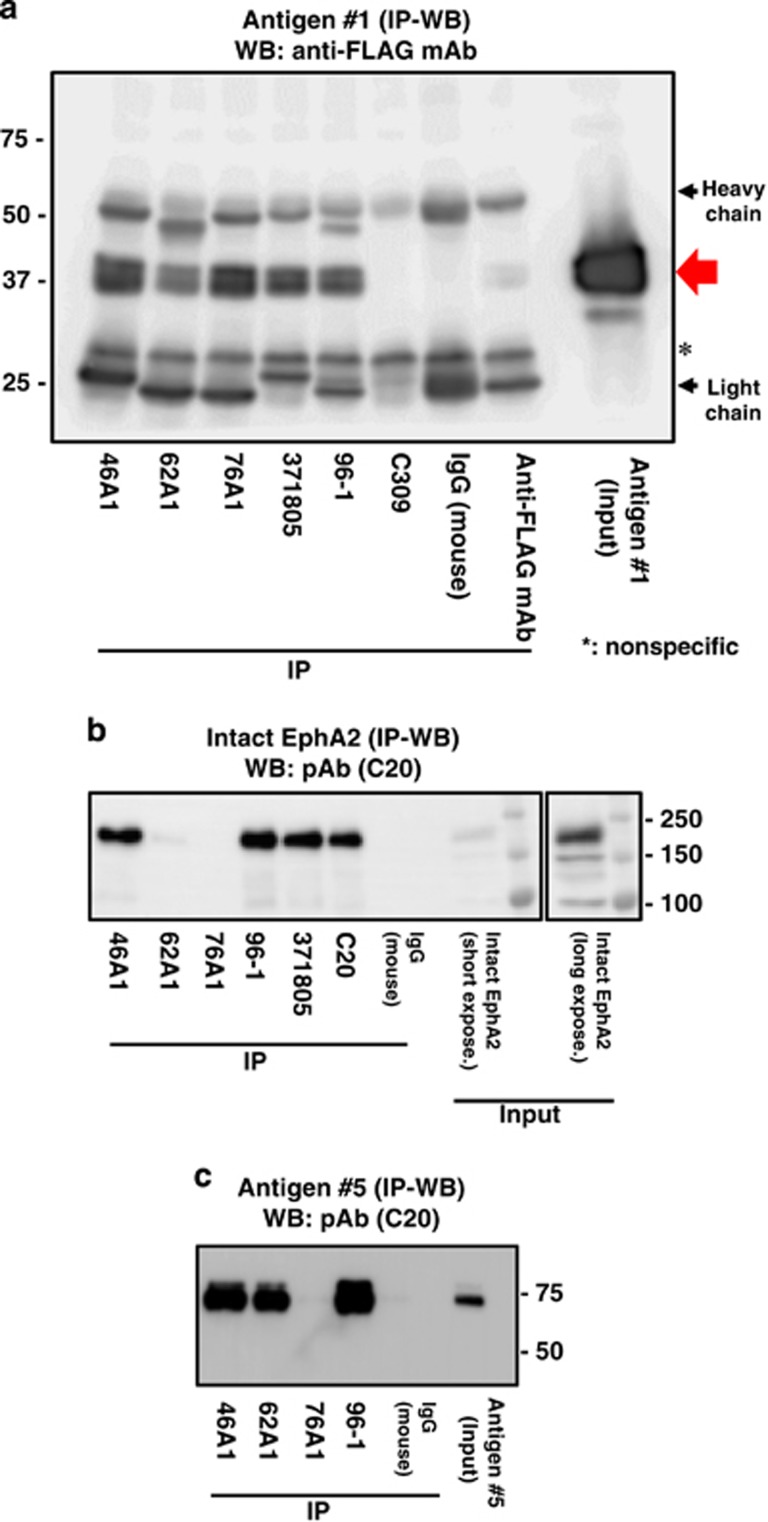
Immunoprecipitation of recombinant EphA2 domains and intact EphA2 protein with anti-EphA2 mAbs. EphA2 proteins expressed in cells were immunoprecipitated by the indicated mAbs. (**a**) The precipitated 37 kDa band corresponding to antigen #1 was detected by western blot using an anti-FLAG mAb (red arrow). This band was recognized by mAbs 46A1, 62A1, and 76A1 immunized by antigen #1. Antigen #1 was precipitated weakly by the anti-FLAG mAb. Immunoglobulins (heavy and light chains) are indicated by black arrows. *Indicates a 30 kDa nonspecific band. (**b**) Precipitated intact EphA2 in the A431 cell lysate was detected by western blot using an anti-EphA2 cytoplasmic domain pAb (C20). mAb 46A1 and positive control antibodies (96-1, 371805, and C20) reacted with intact EphA2. mAb 62A1 recognized intact EphA2 very faintly. In contrast, mAb 76A1 did not show any positive signal. The right panel shows a long exposure to intact EphA2 detected by pAb C20. (**c**) Precipitated antigen #5 (extracellular domain of EphA2) was detected by western blot using an anti-EphA2 cytoplasmic domain polyclonal antibody (pAb) (C20). mAb 46A1, 62A1 and positive control antibody (96-1) reacted with antigen #5, but mAb 76A1 did not. IP, immunoprecipitation; WB, western blot

Mice were immunized with antigen #1 (5 *μ*g/mouse) and 400 fused cells were obtained. The recovered mAbs were screened by ELISA using plates coated with antigen #1 (0.5 *μ*g/plate). Three different hybridoma clones (46A1, 62A1, and 76A1) that produced the IgG_1_ class of mAbs were selected and analyzed further. To confirm reactivity of the mAbs, antigen #1 was immunoprecipitated using them, or other available anti-EphA2 antibodies, and was subjected to 12.5% sodium dodecyl sulfate polyacrylamide gel electrophoresis (SDS-PAGE) under reducing conditions. This was followed by western blot analysis using an anti-FLAG mAb (Figure 2a). All three mAbs precipitated antigen #1 similarly to the positive control antibodies (371805 and 96-1); however, negative control antibodies (mouse IgG and anti-FLAG mAb) did not precipitate antigen #1 (Figure 1a).

To map their epitopes, the four recombinant EphA2 fragments (Figure 1a, antigens #1–4) were subjected to SDS-PAGE under reducing or non-reducing conditions and were analyzed with western blot analysis (Supplementary Figure S1). mAbs 62A1 and 76A1 detected antigen #1 under reducing conditions (Figure 2a), whereas mAb 46A1 failed to detect this antigen in non-reducing conditions (Supplementary Figure S1). mAb 62A1 reacted with antigen #3, but the two other mAbs did not react with antigens #2, 3, or 4. This indicates that epitopes of the three new mAbs exist in an area of antigen #1 that does not overlap with antigens #2, 3, or 4.

To confirm reactivity of the mAbs to other forms of EphA2 carrying the reactive epitope, the entire extracellular domain (antigen #5) was expressed in human epidermoid carcinoma A431 cells that also expressed endogenous EphA2. The cells were lysed and subjected to immunoprecipitation with the mAbs, and then western blotted under non-reducing conditions. Positive control antibodies (96-1, 371805, and C20) immunoprecipitated the intact form effectively, and the protein was detected by western blot analysis using the C20 mAb (Figure 2b). Interestingly, whereas mAb 46A1 recognized the intact form, mAb 76-1 did not and 62A1 did so only weakly. It is of note that mAb 76A1 also failed to recognize the whole EphA2 extracellular domain fragment (antigen #5; Figure 2c). Therefore, mAb 76A1 appears to recognize the soluble EphA2 fragment in a fragment-specific manner rather than the intact and whole extracellular domain-containing forms. In contrast, mAbs 46A1 and 62A1 reacted with all EphA2 forms containing its epitope (intact EphA2, antigens #1 and 5) and mAb 62A1 recognized antigens #1 and #5 (Figures 2a and c). On the basis of these characterizations, mAb 76A1 is the most suitable to capture the soluble EphA2 fragment in blood and to develop a sandwich ELISA system as a diagnostic tool.

### Analysis of the soluble EphA2 fragment in sera of cancer patients and healthy volunteers

**Figure 3:**
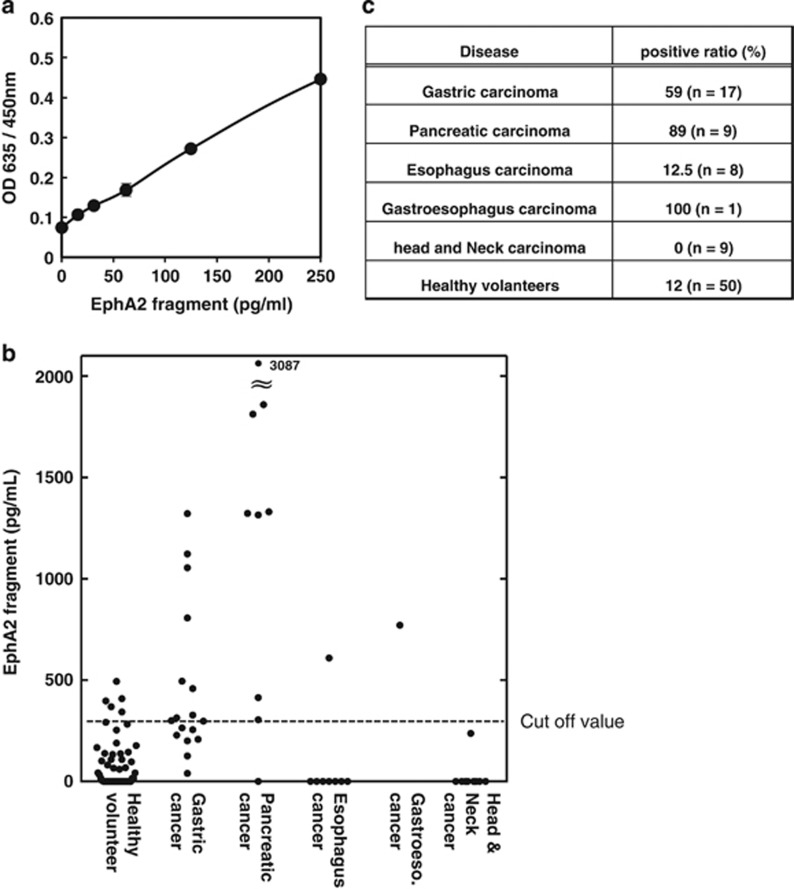
Sandwich ELISA. A sandwich ELISA using a combination of the mAb 76A1 as a capture antibody, and mAb 62A1 for detection was developed and used for this assay. (**a**) Standard curve for detecting an increasing amount of the soluble EphA2 fragment using antigen #1 (0–250 pg/ml). (**b**) Concentrations of soluble EphA2 fragments in serum specimens from carcinoma patients and healthy donors. The cutoff value of 350 pg/ml was determined as the mean plus two S.D.s. (**c**) Positive ratios of serum specimens obtained from patients with different carcinomas and healthy donors

A combination of two mAbs (76A1 and 46A1) was used for a sandwich ELISA; 76A1 to capture antigen #1 and 46A1 for detection. To examine the quantitative reactivity of this ELISA, we added increasing amounts of antigen #1 (0–250 pg/ml) to the test sera. The standard curve exhibited concentration-dependent linearity (Figure 3a). Reactivity did not saturate at least up to 10 ng/ml (data not shown). We then applied this method to analyze 94 serum samples from 17 gastric, nine pancreatic, eight esophageal, one gastroesophageal, and nine head-and-neck carcinoma patients, and 50 healthy donors (26 men and 24 women; Figures 3b and c). The average soluble EphA2 fragment concentration obtained from healthy donors was 96±130 pg/ml. Higher concentrations of the soluble EphA2 fragment were detected in patients with gastric carcinoma (460±379 pg/ml) and pancreatic carcinoma (951±317 pg/ml). On the basis of average values of the healthy donors, the cutoff value was set at 350 pg/ml (mean plus two S.D.s) to predict cancer from patient sera. It is of note that eight out of nine pancreatic cancer patients exhibited soluble EphA2 fragment levels over the cutoff value (Figures 3b and c and Supplementary Table).

**Figure 4:**
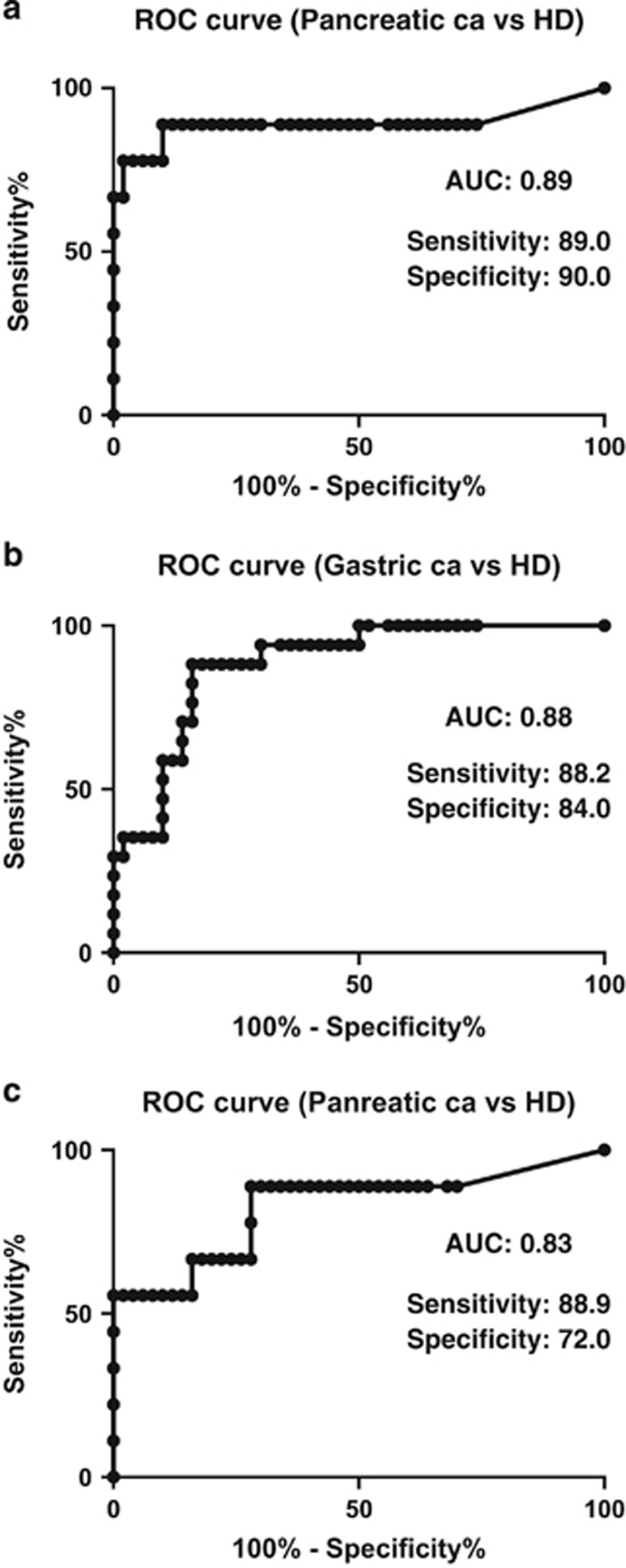
ROC curves. ROC curves associated with soluble EphA2 fragments in terms of detecting (**a**) pancreatic cancer (nine patients), (**b**) gastric cancer (17 patients), and healthy donors (HDs; 26 men and 24 women). (**c**) ROC curve associated with CA19-9 in terms of detecting pancreatic cancer (nine patients) and HDs

Preliminary receiver operating characteristic curve analysis demonstrated that the area under the curve ratios for detecting the soluble EphA2 fragment in pancreatic and gastric cancer patients were 0.89 and 0.88, respectively. The sensitivity and specificity for pancreatic cancer *versus* healthy donors were 89.0% and 90.0%, respectively; *P*<0.0002 (Figure 4a), and for gastric cancer were 88.2% and 84.0%, respectively; *P*<0.0001 (Figure 4b). For comparison, we also measured values of CA19-9 as a conventional biomarker for digestive tumors, including pancreatic cancer (Figure 4c). The sensitivity and specificity of CA19-9 detection in sera from pancreatic cancer patients were 88.9% and 72.0%, respectively; *P*<0.0002. The area under the curve ratio of CA19-9 detection in sera from pancreatic cancer patients was 0.83. These findings suggest that the diagnostic accuracy of measuring levels of the soluble EphA2 fragment specific to pancreatic cancer exceeds that of CA19-9 (Figures 4a and c).

## Discussion

In normal tissues, EphA2 is expressed mainly in epithelial cells where MT1-MMP is not expressed. However, MT1-MMP is usually expressed in activated stromal cells, such as myofibroblasts, invading endothelial cells, and monocytes.^19, 20, 21^ Furthermore, MT1-MMP is frequently overexpressed in cancer cells together with EphA2 as reported in many previous studies and in the Cancer Genome Atlas database (http://cancergenome.nih.gov/). Expression of both proteins has been implicated in malignant behavior including aggressive growth, invasion, and metastasis of cancer cells.^3, 6, 14^ Notably, EphA2 is a substrate of MT1-MMP and can be processed on the surface of cancer cells.^9, 18^ Proteolytic processing of EphA2 by MT1-MMP eliminates the EphA2 ligand-binding domain and converts it from a ligand-dependent tumor suppressor to a ligand-independent oncoprotein.^9^ Therefore, the proteolytic processing of EphA2 by MT1-MMP represents a cancer cell-specific event. This implies that the products of EphA2 cleavage could constitute markers for cancer progression.

To establish a method for detecting the products of EphA2 processing in cancer patients, we developed mAbs specific to the extracellular domain of EphA2 (antigen #1). One of the three mAbs we developed, mAb 76A1, reacted specifically with the soluble EphA2 fragment, with negligible cross-reactivity to the intact form (Figure 2). A quantitative sandwich ELISA was developed by taking the advantage of the specificity of the 76A1 mAb. We then applied this ELISA to commercially available serum specimens obtained from cancer patients and healthy donors. Higher levels of the soluble EphA2 fragment were observed in cancer patient-derived sera compared with healthy donors. In particular, the levels for pancreatic and gastric cancers were significantly higher than the cutoff values, although this was not the case for esophageal and head-and-neck carcinoma samples. This suggests that, in addition to the expression of MT1-MMP and EphA2, other factors could be involved in releasing the EphA2 fragment into blood vessels from cancer cells.

Even though this analysis was preliminary in terms of the number of patients and the potential to improve the sensitivity of the ELISA, it was clearly evident that the soluble EphA2 fragment exists in blood from cancer patients and could be useful as a diagnostic biomarker for detecting pancreatic cancer. Although CA19-9 is frequently used for a clinical blood test to screen for pancreatic cancer,^22, 23^ the soluble EphA2 fragment appears to be more accurate and has the potential to replace CA19-9 or be used in a complementary manner to overcome the limitations of CA19-9.

EphA2 includes both intact and cleaved forms secreted from cancer cells.^9, 18, 24^ In our preliminary results, high levels of EphA2 were detected frequently in serum from healthy donors (382±493 pg/ml, *n*=26) when we used mAb 46A1 that reacts with both soluble EphA2 fragments and the intact form (Supplementary Figure S2), whereas EphA2 values detected by mAb 76A1 were almost negligible (Supplementary Figure S2). These results suggest that both EphA2 forms may exist in normal sera from healthy donors and, presumably, sera from cancer patients. It is likely that various EphA2 forms from non-cancerous cells diminish the sensitivity and specificity of that assay as a diagnostic tool for cancer patients.

On the basis of the results of our present study, we propose that the soluble EphA2 fragments shed into blood are a valuable biomarker for the presence of cancer cells in patients, particularly in pancreatic carcinoma. The development of mAbs that specifically recognize the soluble forms of EphA2 with negligible cross-reactivity to the intact forms is the key for accurate diagnosis. Although the number of patients subjected to this assay in our study was not large, the present results are sufficiently positive for testing a larger number of patient specimens using this assay method. We are improving the detection system further as a diagnostic tool and planning its evaluation in a larger patient cohort. Overall, specific detection of the soluble EphA2 fragment cleaved by MT1-MMP in cancer cells is likely to become a key technology of pancreatic carcinoma diagnosis using blood specimens.

## Materials and methods

### EphA2 antibodies

The anti-EphA2 mAb 96-1 and polyclonal antibody (pAb) C309 were gifts from Daiichi Sankyo (Tokyo, Japan). The epitopes of mAbs 96-1 and pAb C309 were determined to be a ligand-binding and cysteine-rich domain, respectively. pAbs 371805 and C20 against EphA2 were purchased from R&D Systems (Minneapolis, MN, USA) and Santa Cruz Biotechnology (Dallas, TX, USA), respectively. Epitopes of the pAbs 371805 and C20 consisted of EphA2 ligand-binding and cytoplasmic domains, respectively. All antibodies were applicable for immunoprecipitation assays, except pAb 309.

### Recombinant antigens

Human EphA2 cDNA was purchased from Open Biosystems (Huntsville, AL, USA). Gene fragments corresponding to nucleotides 1–981 and 1–1578 of the coding region (antigens #1 and 5) were amplified using the polymerase chain reaction, fused to a FLAG-tag-coding sequence at the 3′ end, and subcloned into the pcDNA3.2-DEST mammalian-expression vector using the Gateway System (Invitrogen, Carlsbad, CA, USA; Figure 1). Expression vectors for the EphA2 ligand-binding and cysteine-rich domains (antigen #1) and EphA2 extracellular domain (antigen #5) were transiently transfected under serum-free conditions into FreeStyle 293-F and A431 cells, respectively. These recombinant antigens were secreted into serum-free conditioned medium, collected, and purified by anti-FLAG mAb-conjugated agarose-affinity chromatography as described previously.^25^ Purity of the recombinant proteins was verified by SDS-PAGE with Coomassie Brilliant Blue staining.

For antibody epitope mapping, antigens #2, 3, and 4, corresponding to EphA2 aa 28–125, 101–250, and 226–328, respectively, with C-terminal FLAG tags, were amplified using the polymerase chain reaction and subcloned into the pET160 vector using the Gateway System (Invitrogen; Figure 1). Recombinant antigens were expressed in *E. coli* and purified with anti-FLAG mAb-conjugated agarose chromatography as described previously.^21^

### Immunization, hybridization, and hybridoma screening

BALB/c mice were injected intraperitoneally with 50 *μ*g of recombinant soluble EphA2 fragments containing the ligand-binding domain (antigen #1) and emulsified in an equal volume of complete Freund’s adjuvant (Difco, Sparks, MD, USA). Immunization was boosted twice at 2-week intervals with equal amounts of antigen #1 with incomplete Freund’s adjuvant (Difco). Six weeks after the third immunization, mice were injected intravenously with the same amount of antigen #1 without adjuvant. Three days after this injection, blood was collected and centrifuged at 1500 × *g* at room temperature to obtain serum for use as a positive control (that is, containing antibodies specific to antigen #1) during hybridoma screening.

Hybridization of the P3U1 mouse myeloma cell line and spleen cells was performed according to methods described by IBL (Gunma, Japan). Hybridoma cells were cultured in HAT selection medium for 10 days, and the cells were re-seeded into 96-well plates. Supernatants from each well containing growing cells were examined for antibody production using antigen #1 and an ELISA. Limited dilution and cloning of cells was repeated twice. Three hybridoma clones producing mAbs reactive to the soluble EphA2 fragment (mAbs 46A1, 62A1, and 76A1) were obtained. To analyze the epitopes of these mAbs, western blotting using recombinant antigens #2, 3, and 4 was performed under non-reducing and reducing conditions.

### Immunoprecipitation

The soluble EphA2 fragment (antigen #1; 1 *μ*g), uncleaved extracellular domain of EphA2 (antigen #5; 1 *μ*g), or intact EphA2 from the A431 cell lysate were incubated overnight at 4 °C with mAbs or pAbs in radioimmunoprecipitation assay buffer (final volume 200 *μ*l). Forty microliters of suspended Protein-G magnetic beads were added and incubated at 4 °C for 1 h with agitation. The beads were washed three times with radioimmunoprecipitation assay buffer and those containing antigen/antibody complexes were removed with a magnetic rack. Precipitated antigen–antibody complexes were released from the beads by addition of 2 × sodium dodecyl sulfate sample buffer containing 5% (v/v) *β*2-mercaptoethanol. Details are described in our previous report.^25^

### Sandwich ELISA

The 96-well plates were coated with mAb 76A1 or 46A1 (20 *μ*g/ml) in phosphate-buffered saline overnight at 4 °C. Pre-immune mouse serum was used as the negative control. The wells were blocked with protein-free blocking buffer (Pierce Biotechnology, Rockford, IL, USA) for 1 h and dried for 40 min at room temperature. Each well was washed three times with phosphate-buffered saline containing 0.05% Tween-20, followed by adding 0–1000 pg/ml of the MT1-MMP-cleaved fragment (antigen #1) to the wells for reaction with biotin-conjugated mAb 62A1 for 2 h at 37 °C. After washing, bound antigens were detected with horseradish peroxidase-labeled Avidin-D (0.1 *μ*g/ml). The absorbance/reference (590/620 nm) of the wells filled with 3,3',5,5'-tetramethylbenzidine, after stopping the reaction with 1 N H_2_SO_4_, was measured using an iMark microplate reader (Bio-Rad, Hercules, CA, USA). Detailed methods were described previously.^26^

### Clinical specimens

Pancreatic carcinoma sera (nine cases) were obtained from ProMedDx (Norton, MA, USA). Gastric (17 cases), esophageal (8 cases), gastroesophageal (1 case), and head-and-neck (9 cases) cancer sera, and sera from 50 healthy donors (26 men and 24 women) were purchased from Kokusai Bio (Tokyo, Japan). Information about serum specimens is shown in the Supplementary Table. All serum specimens were used after centrifugation at 1500 × *g* for 15 min at 4 °C. Levels of the conventional digestive tumor marker, CA19-9, were measured using a chemiluminescence immunoassay in serum specimens from cancer patients and healthy donors.^23^

### Statistical analysis

Data were analyzed using GraphPad Prism 6 software (GraphPad Software, La Jolla, CA, USA). A *P*-value of <0.05 was considered statistically significant by the Mann–Whitney *U*-test.

## Publisher’s Note

Springer Nature remains neutral with regard to jurisdictional claims in published maps and institutional affiliations.
