# Supplementary material for: Specific detection of soluble EphA2 fragments in blood as a new biomarker for pancreatic cancer
Source: Cell Death Dis. 2017 Oct 26;8(10):e3134–. doi: 10.1038/cddis.2017.545 (PMC5680914; doi:10.1038/cddis.2017.545)
Supplement: Supplementary Figure 1 [file cddis2017545x1.pdf]

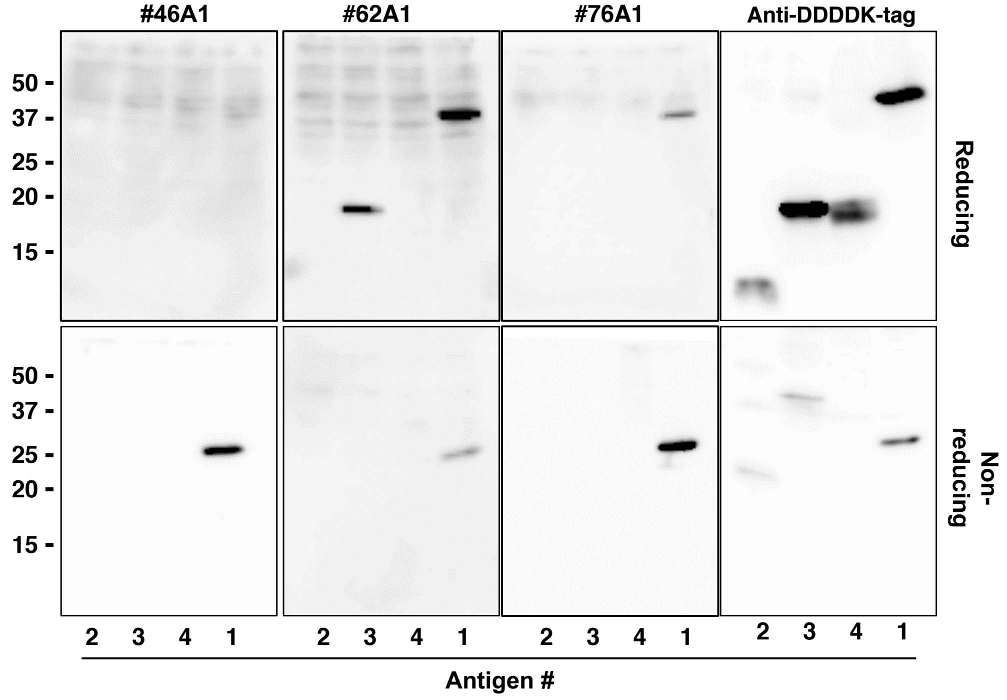

Antigen #1: ^{1}M-^{327}R (expressed in HEK293 cells)
Antigen #2 : ^{28}E-^{125}E (expressed in *E.coli*)
Antigen #3 : ^{28}E-^{24}A + ^{101}T-^{250}D (expressed in *E.coli*)
Antigen #4 : ^{28}E-^{24}A + ^{226}A-^{327}R (expressed in *E.coli*)

Figure S1. Epitope mapping of EphA2 antibodies.

Western blot analysis of the affinities of monoclonal antibodies (mAbs) 46A1, 62A1, and 76A1 to recombinant antigens #1–4 under reducing (upper panel) and non-reducing (bottom panel) conditions. All antigens were detected by an anti-FLAG mAb under reducing and non-reducing conditions.
