# Supplementary material for: Specific detection of soluble EphA2 fragments in blood as a new biomarker for pancreatic cancer
Source: Cell Death Dis. 2017 Oct 26;8(10):e3134–. doi: 10.1038/cddis.2017.545 (PMC5680914; doi:10.1038/cddis.2017.545)
Supplement: Supplementary Figure 2 [file cddis2017545x2.pdf]

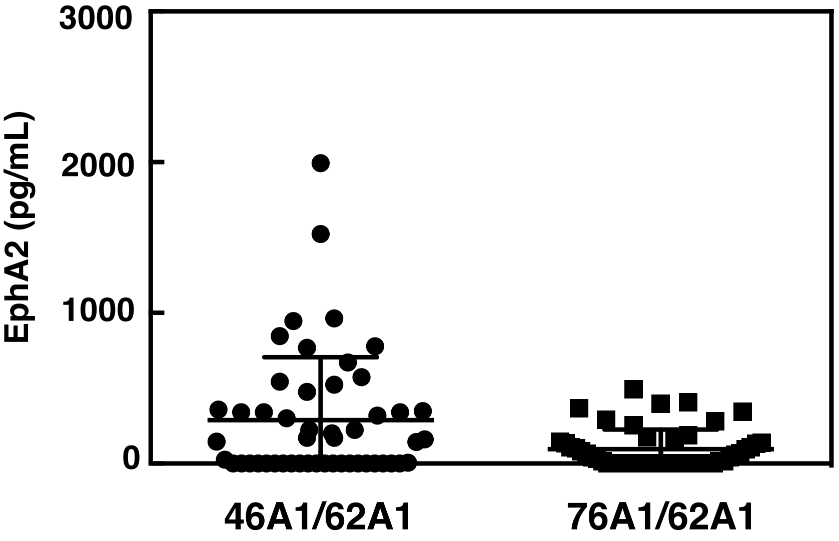

Figure S2. Comparison of serum EphA2 levels in healthy donors.

Sandwich enzyme-linked immunosorbent assay (ELISA) using monoclonal antibody (mAb) 46A1 as the capture antibody and mAb 61A1 for detection yielded some false positive reactions due to detection of both intact and soluble EphA2 fragments (n = 26). In contrast, the sandwich ELISA using mAb 76A1 for capture and mAb 62A1 for detection showed that the standard deviation value for soluble EphA2 fragment in healthy donors was small and tightly gathered around the mean value.
