# Supplementary material for: Specific detection of soluble EphA2 fragments in blood as a new biomarker for pancreatic cancer
Source: Cell Death Dis. 2017 Oct 26;8(10):e3134–. doi: 10.1038/cddis.2017.545 (PMC5680914; doi:10.1038/cddis.2017.545)
Supplement: Supplementary Table 1 [file cddis2017545x3.pdf]

Supplementary Table.

Information of Serum Specimens

Normal Sera

No.	Gender	Age	EphA2 fragment (pg/mL)
#1	M	48	0.0
#2	M	33	101.4
#3	M	30	0.0
#4	M	51	0.0
#5	M	43	0.0
#6	M	43	15.5
#7	M	49	408.7
#8	M	50	65.2
#9	M	61	42.6
#10	M	49	15.5
#11	M	44	138.1
#12	F	30	0.0
#13	M	33	42.0
#14	M	35	0.0
#15	M	40	0.0
#16	M	40	0.0
#17	M	57	0.0
#18	M	49	493.6
#19	M	37	368.7
#20	M	47	0.1
#21	M	47	0.1
#22	M	49	0.1
#23	F	45	0.1
#24	M	44	0.1
#25	F	30	0.1
#26	M	38	0.1
#27	M	45	397.5
#28	M	42	282.2
#29	F	30	291.8
#30	M	33	166.9
#31	M	46	32.4
#32	M	40	0.0
#33	M	45	80.4
#34	M	41	253.4
#35	M	63	3.2
#36	M	52	15.4
#37	M	58	67.9
#38	M	32	96.2
#39	M	43	189.2
#40	F	42	108.4
#41	F	39	177.1
#42	M	44	342.9
#43	M	53	0.0
#44	M	51	0.0
#45	F	30	59.8
#46	M	42	0.0
#47	M	41	136.7
#48	M	54	132.6
#49	M	34	144.7
#50	M	33	108.4

Cancer Sera

Cancer	No.	Stage	Grade	EphA2 fragment (pg/mL)
Gastroeso. Ca	#1	N/A	N/A	125.9
	#2	IA	N/A	327.3
	#3	IA	G2	1321.6
	#4	IA	N/A	1055.0
	#5	IV	G4	208.0
	#6	III	N/A	806.4
	#7	I	G3	300.1
	#8	III	G2	494.5
	#9	IV	G2-3	255.2
	#10	II	G3	39.3
	#11	IIIA	G2-3	228.2
	#12	IB	N/A	297.9
	#13	IIIA	G2	1122.7
	#14	II	G1	264.2
	#15	IIIA	N/A	313.8
	#16	II	G2	458.4
	#17	III	N/A	200.1
Pancreatic Ca	#1	IV	N/A	304.7
	#2	IV	N/A	0.0
	#3	IV	N/A	413.5
	#4	IV	N/A	1315.0
	#5	II	N/A	3087.0
	#6	N/A	N/A	1812.4
	#7	III	N/A	1859.0
	#8	IV	N/A	1330.6
	#9	IV	N/A	1322.8
Esophagus Ca	#1	I	N/A	0.0
	#2	II	N/A	0.0
	#3	IV	N/A	0.0
	#4	IV	N/A	0.0
	#5	II	N/A	0.0
	#6	IV	N/A	0.0
	#7	III	N/A	0.0
	#8	I	N/A	608.9
Gastroeso. Ca	#1	IV	N/A	771.0
Head&Neck Ca	#1	N/A	N/A	0.0
	#2	III	N/A	0.0
	#3	II	N/A	237.3
	#4	N/A	N/A	0.0
	#5	III	N/A	0.0
	#6	N/A	N/A	0.0
	#7	N/A	N/A	0.0
	#8	N/A	N/A	0.0
	#9	N/A	N/A	0.0
